# Thinking Out of the Box: A Green and Social Climate Fund: Comment on "Politics, Power, Poverty and Global Health: Systems and Frames"

**DOI:** 10.15171/ijhpm.2016.154

**Published:** 2016-12-28

**Authors:** Gorik Ooms, Remco van de Pas, Kristof Decoster, Rachel Hammonds

**Affiliations:** ^1^ Department of Global Health and Development, London School of Hygiene and Tropical Medicine, London, UK.; ^2^ Department of Public Health, Institute of Tropical Medicine, Antwerp, Belgium.; ^3^ Law and Development Research Group, Faculty of Law, University of Antwerp, Antwerp, Belgium.

**Keywords:** Global/Planetary Health, Inequality, Politics, Greenhouse Gas

## Abstract

Solomon Benatar’s paper "Politics, Power, Poverty and Global Health: Systems and Frames" examines the inequitable state of global health challenging readers to extend the discourse on global health beyond conventional boundaries by addressing the interconnectedness of planetary life. Our response explores existing models of international cooperation, assessing how modifying them may achieve the twin goals of ensuring healthy people and planet. First, we address why the inequality reducing post World War II European welfare model, if implemented state-by-state, is unfit for reducing global inequality and respecting environmental boundaries. Second, we argue that to advance beyond the ‘Westphalian,’ human centric thinking integral to global inequality and climate change requires challenging the logic of global economic integration and exploring the politically infeasible. In conclusion, we propose social policy focused changes to the World Trade Organisation (WTO) and a Green and Social Climate Fund, financed by new global greenhouse gas charges, both of which could advance human and planetary health. Recent global political developments may offer a small window of opportunity for out of the box proposals that could be advanced by concerted and united advocacy by global health activists, environmental activists, human rights activists, and trade unions.

Solomon Benatar’s recent paper addressing “Politics, Power, Poverty and Global Health: Systems and Frames”^1^ reminds us of the famous quote (commonly, but probably mistakenly, attributed to Albert Einstein)^2^: “We cannot solve our problems with the same thinking we used when we created them.” We agree with Benatar: as long as we seek solutions to address global health inequalities within the same systems and frames that produced them, we may be unable to find any.

Does the dearth of published scholarly debate providing ‘out of the box’ solutions reveal a lack of imagination or creativity, as Benatar seems to suggest? We do not think so. We spend a fair number of ‘after-hours’ hours rethinking the world. And we assume that many of our fellow global health scholars do. These are quite imaginative and creative discussions. But they happen after-hours, because the academic system provides few incentives to produce ideas that cannot be published as research or readily implemented. So, let us use the space offered by this journal to start sharing and discussing out of the box ideas; starting with how to address growing global inequality to broader questions about the necessity of integrating this response into actions that tackle human impact on the planet.

Anthony Atkinson’s recent book, “Inequality: What can be done?,” is not a book about health inequality, but about economic inequality, with a focus on household income.^3^

However, the work of Michael Marmot^4^ and the Commission on the Social Determinants of Health^5^ reveals the close relationship between economic inequality, social inequality, and health inequality. Atkinson reminds us that we do know how to reduce income inequality: after World War II, the welfare state (including collective bargaining of salaries, which reduced inequality in ‘market incomes,’ and progressive taxation, which allowed inequality-reducing redistribution of market incomes) pushed inequality in Europe downwards substantially. Since the 1980s, however, the United Kingdom, followed by many others, took an ‘inequality turn’: the collective bargaining power of trade unions declined, taxation became less progressive, and social protection policies eroded.^3^

## Three Problems

So, one could argue that we do not have to think out of the box after all; we should simply restore the European welfare state policies of the 1950s to the 1970s, and expand them to the rest of the world. Unfortunately, it is not that simple. There are at least three major problems:

- First, European welfare state policies worked, at least to some extent, within a context of increasing global economic integration, in which businesses located in European (and other high-income) countries enjoyed the benefit of a highly skilled workforce and cheap imported raw commodities. The decreasing inequality within European countries went together with increasing inequality between countries – the gap between the average income in wealthier and poorer countries widened.^6^ But, in terms of average income, many poorer countries are catching up, and investments and jobs are moving towards them – where the workforce is now more skilled than it used to be, and still much cheaper – and away from the wealthier countries. This is why some argue that the European welfare state of the second half of the 20th Century cannot be expanded to other parts of the world.^7^ Atkinson rebuffs this objection against a return to the welfare state, but does not dismiss it altogether: “There is a fiscal problem, but it is a problem that is within our powers to solve, not one whose outcome is determined purely by external forces.”^4^ Increasing international cooperation could, in Atkinson’s opinion, create conditions for the establishment of the welfare state in all countries of the world: avoiding the ‘tax competition’ that erodes government revenue and thus, the space for social policy by creating minimum taxation and social policy standards.
- That brings us to the second problem: the poorer countries may not be keen to engage in the kind of international cooperation that would be required. For decades, they have seen the average income gap between them and wealthier countries widen, and now that the gap is finally starting to decrease, the wealthier countries – or progressive voices in wealthier countries – want them to adopt policies that would reduce their attractiveness to investors? Discourses about the importance of solidarity and lesser inequality within countries, from the same countries that benefitted from rising inequality between countries and never seriously considered redistribution of income across state borders, sound shallow and hollow. Moreover, the same wealthier countries are ambiguous, to say the least, in their position on decent employment, wage bills and fiscal policy in poorer countries. As the main powers governing the post World War II International Financial Institutions, and the drivers of the new generation of regional Free Trade Regimes, wealthier countries are incoherent: they talk about inclusive growth, but at the same time set the global policy for a ‘new Gilded Age.’^8,9^ The kind of international cooperation required to allow all countries to adopt the European welfare state model will have to include at least redistribution between countries.
- Third, the European welfare state model flourished during decades of substantial and continuous economic growth, which allowed policies that did not ‘set back’ any strand of society; these policies only ‘pushed forward’ some strands faster than others. To do something similar at the global level would require global economic growth that would be environmentally unsustainable, and thus, ultimately ineffective.^10^ If a global population of 10 billion people, which is projected for 2050, copies the present consumption habits of the average household of wealthier countries that would have unimaginably negative environmental consequences.^11^ So, we also must change consumption habits radically, promoting environmentally friendly consumption, and discouraging environmentally destructive production and consumption.

## Three out of the Box Solutions

How might we start to overcome these three problems? Allowing ourselves the freedom to think out of the box and to disregard the political feasibility of our ideas, we propose the following:

- First, the World Trade Organization (WTO) should require “decent” social policy standards as a condition for becoming, or remaining, a WTO member.^12^ At a minimum, decent standards would include the ratification and implementation of the eight ‘fundamental’ International Labour Organization (ILO) conventions,^13^ and an additional one, inspired by the ILO social protection floor.^14^ The latter could stipulate, for example, that public social expenditure should reach at least the equivalent of 24% of gross domestic product (GDP) in high-income countries; the equivalent of 12% of GDP in low-income countries; and provide a sliding scale between 12% and 24% for middle-income countries. While no country would be forced to adopt these standards, the price of not adopting them would be exclusion from the WTO.
- Second, the United Nations (UN) should create a global social fund, collecting and redistributing US$800 billion per year, or the equivalent of 1% of the global GDP. This amount would be administered by different smaller funds: one for health, one for education, one for child support, one for pensions, and so on.
- Third, an annual global greenhouse gas auction, as proposed by Simon Caney,^15^ or a global carbon tax, would raise the $800 billion needed to finance the global social fund. All businesses – from large transnational corporations to small family-run businesses – would have to buy rights to emit greenhouse gasses; only individual persons, up to a limited threshold, would be exempted. To encourage a decline in average income inequality between countries, production units in below average emission countries would benefit from an agreed discount. This would make goods and services that harm the environment more expensive – or should we say: as costly as they really are? – and environmentally friendly goods and services cheaper.

Technically, all of this is feasible. We know how to monitor the implementation of social policy standards. We know how to exclude countries from the WTO. We know how to create and administer global funds. And we know how to organise greenhouse gas auctions or a carbon tax.

The political feasibility is a different matter: this is not the same kind of thinking as that which created the problems of inequality and climate change. This thinking is taking giant steps away from the ‘Westphalian’ world order – not baby steps. And yes, we concur that the political tide is moving, unfortunately, in a different direction, as evidenced by the increasing nationalism in Europe and the election of an American President with isolationist views. The role of increasing inequality in these results is underlined by Thomas Piketty who argues that “Trump’s victory is primarily due to the explosion in economic and geographic inequality in the United States over several decades and the inability of successive governments to deal with this.”^16^ In addition, there is a profound gridlock in international cooperation and global governance that is not easily resolved. Powerful nation states, by and large, move away from multilateral cooperation and sharing the responsibilities for the global economic, demographic, and environmental risks we face, except perhaps when it comes to governing the urgent challenge of climate change.^17^ However, these political developments may present a window of opportunity. While increasing nationalism and Trump’s election may be pushing the window closed, conversely, they may be pushing it open as people may now mobilise to avert what Benatar terms, “the tragedies visible on the horizon that are already becoming manifest.”^1^ However, any window that is opening will not remain open for long and once it is closed it is likely to remain closed for some time.

Nevertheless, our proposals are perhaps not so out of the box as they may appear. The WTO exists, even though it is criticized for promoting a kind of global economic integration that allows and encourages countries to sacrifice social policy standards – and we concur with the critiques. Moreover, analyses of regional Free Trade Regimes in advanced stages of development such as the Trans-Pacific Partnership (TPP), the Transatlantic Trade and Investment Partnership (TTIP) and the Trade in Services Agreement (TiSA) indicate that domestic ‘regulatory chills’ and international arbitration systems have considerable potential to undermine labour and environmental rights and pose risks for public health and human well-being.^18^ However, as Sarah Joseph argues, “social dumping also constitutes unfair trade, which should therefore justify analogous countermeasures,”^19^ that is, countermeasures analogous to the ones that are allowed in cases of ‘ordinary dumping’ or exporting goods at less than their real cost. A ‘labour rights clause’ would enable the WTO to allow its members, including in regional Free Trade Agreements, to protect themselves from social dumping: “Such protection could take the form of a minimum standards clause, performing a similar function to TRIPS regarding intellectual property protection.”^19^

Both the Green Climate Fund and the Global Fund to fight AIDS, tuberculosis and malaria (Global Fund) exist. The Green Climate Fund is charged with being an ‘empty shell’^20^; the Global Fund is criticized for focusing on three infectious diseases, and if it ever became a global fund for health, it would probably be blamed for ignoring the social determinants of health.^21^ But their very existence allows us to imagine how they could evolve towards a Green and Social Climate Fund.

Several ‘cap-and-trade’ systems for greenhouse gasses exist,^22^ and several states have adopted a carbon tax.^23^ The proceeds are disappointing, and so is their impact on reducing the emission of greenhouse gasses. Again, their very existence allows us to imagine an improved and global version, which could help finance a Green and Social Climate Fund.

The underperformance of existing efforts does not allow a Panglossian ‘all is for the best in the best of all possible worlds’ attitude. But they should not blind us from seeing their potential, if linked together within a vision aiming at addressing inequality and climate change, which Piketty terms “the main challenges of our times.”^15^ Our proposals, social policy focused changes to the WTO and a Green and Social Climate Fund, financed by new global greenhouse gas charges, could advance both human and planetary health. Seizing the small window of opportunity that exists to advance on these intertwined objectives will require concerted and united advocacy by global health activists, environment activists, human rights activists, and trade unions. That may, however, require some real out of the box thinking.

## Ethical issues

Not applicable.

## Competing interests

Authors declare that they have no competing interests.

## Authors’ contributions

This paper is the result of many discussions among the four authors. GO wrote the first draft of the manuscript. RvdP, KD, and RH contributed to the writing of the final manuscript.

## Authors’ affiliations

^1^Department of Global Health and Development, London School of Hygiene and Tropical Medicine, London, UK. ^2^Department of Public Health, Institute of Tropical Medicine, Antwerp, Belgium. ^3^Law and Development Research Group, Faculty of Law, University of Antwerp, Antwerp, Belgium.
